# Similar short-term outcomes for bone-level implants with or without intermediate abutments

**DOI:** 10.1038/s41432-024-01065-9

**Published:** 2024-09-18

**Authors:** Kelvin I. Afrashtehfar, Carlos A. Jurado, Shatha S. R. H. Alnuaimi, Sultan M. S. Alhadhrami

**Affiliations:** 1 Department of Reconstructive Dentistry and Gerodontology, School of Dental Medicine, University of Bern, Bern, Switzerland; 2 Private Practice, Abu Dhabi City, UAE; 3 College of Dentistry, Ajman University, Ajman, UAE; 4 Department of General Dentistry, College of Dentistry, The University of Tennessee Health Science Center, Memphis, TN USA; 5 Division of Operative Dentistry, College of Dentistry, The University of Tennessee Health Science Center, Memphis, TN USA; 6 Bachelor of Dental Surgery Program, College of Dentistry, Ajman University, Ajman City, UAE; 7 Dental Clinic & Dentistry Department, Healthpoint Hospital, M42 Health, Abu Dhabi City, UAE; 8 National Service Recruit, National Service and Reserve Authority (NSRA), Dental Department, Zayed Military Hospital, Abu Dhabi City, UAE

**Keywords:** Dental implants, Peri-implantitis

## Abstract

**Design:**

A single-center (university-setting), prospective, longitudinal, split-mouth, single-blind, randomized controlled clinical trial investigated peri-implant parameters of bone-level implants restored with either screw-retained prostheses connected directly to the implants or with intermediate abutments over a 3-year period. The study adhered to the ethical principles of the Helsinki Declaration and the CONSORT guidelines. Ethical approval was granted, and the trial was registered at Clinicaltrials.gov.

**Case selection:**

Participants included were over 18 years of age, had a plaque index below 25%, and were missing at least two adjacent teeth, allowing for rehabilitation with screw-retained fixed partial prostheses over two implants and 2–4 prosthetic units. Exclusion criteria included long-term use of medications affecting bone metabolism, smoking more than 10 cigarettes per day, history of local radiotherapy, untreated periodontitis, and the need for rehabilitation in the anterior sextant of the maxilla. The primary clinical outcome was marginal bone loss (MBL), while secondary outcomes included probing pocket depth (PPD), plaque index (PI), bleeding on probing (BOP), and patient-reported outcomes (PROs).

**Study timeline:**

The study schedule included a screening (visit 1), implant surgery (visit 2), stage-two 8 weeks post-surgery (visit 3), impressions taken 4 weeks post stage-two (visit 4), baseline standardized radiograph (visit 5), followed by 6-month (visit 6), 12-month (visit 7), and 36-month (visit 8) follow-up visits.

**Data analysis:**

Descriptive statistics and quantitative measures included means, standard deviations (SDs), minimum and maximum values, and 95% confidence intervals (CIs). Clinical parameters (six sites per implant) measured were MBL, PPD, PI, and BOP. Paired *t* tests were utilized for intragroup comparisons across different time points and intergroup comparisons at each time point. PROs at 36 months were compared using Student’s *t* test. The alpha significance level was set at 0.05.

**Results:**

The study included 36 patients (72 implants), with two not completing the follow-up due to death and relocation. No implants showed signs of inflammation or mobility. Mean interproximal bone level (IBL) at baseline was 0.13 mm ± 0.15 mm for the control group and 0.10 ± 0.13 mm for the test group. At the 36-month follow-up, mean IBL was 0.13 ± 0.18 mm for the control group and 0.20 ± 0.24 mm for the test group, with no significant differences (F(1, 32) = 1.06; p > 0.05). Clinical parameters (PPD, BOP, PI) at 36 months showed no significant differences between groups. Minor complications occurred in 6.7% of the control group and 5.3% of the test group. PROs indicated no significant differences in general satisfaction, esthetics, comfort, phonetics, and masticatory function between the groups.

**Conclusions:**

After a 36-month follow-up, bone-level implants restored with CAD/CAM prostheses directly connected to the implants displayed similar clinical outcomes, PROs, and marginal bone level changes as those restored with intermediate standardized abutments.

## A Commentary on

**Maceiras L, Liñares A, Nóvoa L, Batalla P, Mareque S, Pérez J, Blanco J.**

Marginal changes at bone-level implants supporting dental prostheses with or without intermediate standardized abutments after 36 months: Randomized controlled clinical trial. *Clin Oral Implants Res* 2024; 10.1111/clr.14297.

**GRADE Rating**: 

## Commentary

The use of standardized abutments between fixed dental prostheses and implants has been recommended to preserve marginal bone levels and prevent peri-implant disease^1,2^. However, factors like the implant-abutment interface (micro-gap)^3^ and the repeated connection/disconnection of abutments^4,5^ can affect peri-implant bone levels and the establishment of the biological width. Additionally, micro-gaps at the implant-abutment interface can lead to bacterial infiltration and apical migration of the bone level^6^. Therefore, standardized abutments are suggested to distance the micro-gap from the implant shoulder, allowing for biological space formation^7^.

Regarding prosthesis macro design, some studies indicate that wide emergence angles are associated with peri-implantitis in bone-level implants^8^, (while others find no correlation with peri-implant health^9^. Recent research shows mixed results on peri-implant bone loss when comparing prostheses connected directly to implants versus using intermediate abutments^10,11^. Most of these studies had relatively short follow-up periods, except for a recent study^12^ that followed participants for 5 years. Thus, the reviewed randomized control trial (RCT) by Maceiras et al.^13^ evaluates the changes in marginal bone levels in bone-level implants restored with screw-retained prostheses, either connected directly to the implants or with an intermediate abutment, after a 3-year follow-up.

The RCT demonstrates several primary strengths that supports its reliability and validity. For instance, the study design adhered to CONSORT guidelines and received ethical approval. The randomization and split-mouth design minimized selection bias and allowed for direct intra-patient comparisons. The blinding of patients, health workers, and study personnel further reduced potential biases. The study’s inclusion criteria were well-defined, focusing on a specific patient population, which supports the applicability of the results to similar clinical scenarios.

The study’s standardized procedures for implant placement and follow-up assessments ensure reproducibility. Using advanced imaging techniques like cone-beam computed tomography (CBCT) and standardized radiographic methods provided accurate measurements of marginal bone levels. A comprehensive statistical analysis, including mixed ANOVA and reliability analysis, was conducted. By considering both clinical outcomes and patient-reported outcomes (PROs), the study offered a holistic view of the treatment’s impact. Notably, there were no significant differences in marginal bone loss (MBL), clinical variables, or PROs between direct connection and intermediate abutment groups, indicating both methods are viable for screw-retained implant restoration. The study also addressed potential limitations, such as sample size and follow-up duration.

This RCT has several limitations that contribute to its low GRADE (Grading of Recommendations, Assessment, Development, and Evaluations) rating. The 36-month follow-up period, while moderately lengthy, may not be adequate to capture long-term effects on marginal bone levels and peri-implant health. The small sample size of 36 patients reduces the study’s statistical power, and patient dropouts, with two not completing follow-up, could introduce bias. The single-center design at a university limits the generalizability of the findings to more diverse populations. Variability in implant placement and patient characteristics, such as the distribution of posterior implants, may have influenced the results, and potential confounding variables were not fully  controlled. Additionally, the study’s sponsorship by Ticare, Mozo-Grau S.A., and the involvement of the Principal Investigator in grant research with Ticare through the University of Santiago de Compostela, may raise concerns about potential conflicts of interest.

For future research, it is advised to include larger sample sizes and extended follow-up periods to evaluate the long-term effects of various implant restoration techniques on marginal bone levels and peri-implant health. Multi-center trials could improve the generalizability of the findings. Studies should also control for more confounding variables, such as differences in implant placement and patient-specific factors. Additionally, incorporating detailed PROs and examining different prosthesis designs and materials would offer deeper insights into factors affecting implant success. Utilizing advanced imaging techniques and standardized protocols for radiographic assessments would further improve measurement accuracy and consistency, leading to more definitive conclusions.

To sum up, this study found no significant differences in marginal bone loss, clinical variables, or patient-reported outcomes between implants with direct connections and those with intermediate abutments after 36 months. However, the study’s limitations, including a relatively short follow-up period, small sample size, and single-center design, should be considered. Future research is needed to better understand the long-term impacts of implant restoration modalities.

Practice points

- Both direct connection and intermediate abutment methods for implant restoration show comparable outcomes in terms of marginal bone loss and patient satisfaction over a 36-month period.
- Clinicians should consider both restoration methods viable options, but longer follow-up and larger studies are needed for definitive guidance.
